# For Cold in the Head

**Published:** 1892-02

**Authors:** 


### FOR COLD IN THE HEAD.

There is no better remedy than gelsemium. One good, large dose, say ten minims of the fluid extract, taken upon going to bed, will effectually dispose of this troublesome and uncomfortable affection. One dose is usually sufficient.—*Med Summary.*

